# Comprehensiveness, quality, and readability of non-invasive prenatal testing information on Japanese medical institution websites

**DOI:** 10.1016/j.pecinn.2026.100462

**Published:** 2026-02-22

**Authors:** Junya Kohari, Tsuyoshi Okuhara, Ritsuko Shirabe

**Affiliations:** a Department of Health Communication, School of Public Health, Graduate School of Medicine, The University of Tokyo, Tokyo, Japan; b University hospital Medical Information Network (UMIN) Center, The University of Tokyo Hospital, Tokyo, Japan

**Keywords:** Non-invasive prenatal testing, Health information, Genetic counseling, Readability, Internet, Shared decision making, DISCERN

## Abstract

**Objective:**

We conducted an analysis of online information on noninvasive prenatal testing (NIPT) provided by Japanese medical institutions recognized by the certification system (certified institutions) and other institutions (non-certified institutions).

**Methods:**

We identify institutional websites from google Japan that used three keywords related to prenatal testing (*N* = 37). Comprehensiveness was assessed using domestic and international guidelines. Quality was measured using the DISCERN instrument, and readability was evaluated using jReadability.

**Results:**

Among certified institutions, the mean comprehensiveness score was 7.36 out of 20, and the mean DISCERN score was 42.6 out of 80, categorized as “fair,” although nearly half were rated “poor” or “very poor.” Most websites required “lower advanced” reading proficiency. Websites from non-certified institutions showed higher comprehensiveness and readability than certified institutions.

**Conclusion:**

NIPT-related information on certified institution in Japan is often insufficient in terms of quality. By contrast, non-certified institution, although ethically problematic, may provide more comprehensive and higher-quality information. Certified institutions should improve the quality and clarity of web-based communication to support patient's decision making.

**Innovation:**

This is the first study to assess the NIPT-related websites in Japanese medical institutions. Critical informational gaps were identified, highlighting the need for trustworthy and readable online resources.

## Highlights

• This is the first study to assess online information about noninvasive prenatal testing (NIPT) provided by medical institutions.
• Certified institutions lack sufficient, high-quality, and readable NIPT information.
• Non-certified institutions were more comprehensive but made ethically questionable claims.
• NIPT content on most institutional websites demanded advanced reading ability.
• Better online communication supports informed, autonomous shared decision-making.

## 1 Introduction

In recent years, individuals increasingly encounter a large volume of health information. To appropriately manage health information, individuals require adequate health literacy, defined as the capacity to obtain, process, and understand basic health information and services necessary for making appropriate health decision-making [1]. Health literacy affects health outcomes through multiple pathways, including communication barriers between patients and healthcare providers [2]. Accordingly, *Healthy People 2030 has emphasized* the concept of organizational health literacy, highlighting the responsibility of health organizations to provide information that matches the health literacy levels of patients and the public [1]. Recent organizational health literacy initiatives have shown that revising institutional websites and patient portals using plain language, user-testing, and context-specific links to educational resources can improve patients' ability to navigate services and understand test results, particularly among those with limited health literacy [[3], [4], [5], [6]]. Healthcare institutions should provide clearer information, which is also important from the perspective of shared decision-making (SDM), where patients and healthcare providers jointly make decisions using the best available evidence [7].

Online health information seeking behavior (HISB) refers to the practice of obtaining health information through the internet and social media platforms. Currently, approximately 50–80% of internet users seek health information online [[8], [9], [10], [11]]. Online resources are appealing due to their immediate accessibility and ability to empower patients to engage more actively in their healthcare decisions [12]. However, HISB also entails risks, as online content may contain inaccurate information or be presented in formats that are difficult to comprehend, thereby limiting its effective use [11,13]. In the field of prenatal testing including non-invasive prenatal testing (NIPT), online information-seeking is widespread [14,15]. NIPT is a prenatal screening that can be conducted during early pregnancy and can detect fetal chromosomal aneuploidies, such as trisomies 21, 18, and 13 [16]. Many pregnant women rely on internet sources when considering prenatal testing, and such information is likely incorporated into clinical patient–physician communication. Prenatal testing is a critical decision-making process concerning the fetus. Ensuring informational sufficiency is therefore essential to support patients' accurate understanding of the testing process. In Japan, most online NIPT-related content originates from medical institutions; therefore, ensuring comprehensiveness, quality and readability of this information is particularly important. Readability is defined as “the determination by systematic formulae of the reading comprehension level required to understand written materials” [17]. The American Medical Association recommends writing health information at approximately a sixth-grade reading level to maximize accessibility. Nevertheless, multiple studies have demonstrated that most online health materials exceed recommended readability thresholds [[18], [19], [20], [21], [22]]. In the context of genetics, prior research has shown that letters communicating genetic test results [23] and online information on hereditary hearing loss [24] require reading skills above recommended levels, potentially impairing patient comprehension. However, the readability of NIPT information published by Japanese medical institutions has not yet been evaluated, despite its direct relevance to patient understanding and the quality of SDM [7].

Genetic counseling is widely used to support SDM in prenatal testing. Although the Japan Society of Obstetrics and Gynecology initially implemented guidelines and a certification system to assure high-quality genetic counseling for NIPT, non-certified institutions have expanded since 2016. Many such institutions do not provide adequate counseling or follow-up, yet patients use them more than certified institutions, partly due to greater logistical convenience, including fewer required visits [[25], [26], [27]]. Internationally, concern has also been expressed over biased or exaggerated commercial information that may heighten anxiety and compromise SDM [[28], [29], [30]]. While previous studies have mainly examined content published by commercial providers or news outlets, research evaluating information directly disseminated by medical institutions remains scarce. Therefore, a systematic assessment of the comprehensiveness, quality, and readability of NIPT-related information provided by medical institutions is essential to understand how online information influences informed decision making and to support effective SDM.

This study aimed to evaluate the quality of information on NIPT provided on each institution's website and discuss the information provision that leads to appropriate decision-making and treatment behavior. This study addresses the following four research questions:

RQ1: What is the comprehensiveness of NIPT-related content on the websites of certified institutions?

RQ2: What is the quality of NIPT-related information from certified institutions on these websites?

RQ3: What is the readability of the NIPT-related content from certified institutions on these websites?

RQ4: What is the comprehensiveness, quality, and readability of NIPT-related content on websites of non-certified institutions?

## 2 Methods

### 2.1 Study design

We conducted a Japanese-language search using Google Japan, the most widely used search engine in Japan [31]. Three search terms were used: “NIPT,” “shusshoumaeshindan” (meaning prenatal diagnosis), and “shusshoumaekensa” (meaning prenatal testing). We entered each keyword individually into the search window, one keyword at a time, and 30 websites were reviewed for each term. This is because most Internet users do not view the first three pages of the search results [32]. To prevent retrieval of restricted pages that were inaccessible to the public, we conducted all searches without logging into any institutional accounts. To minimize search bias, we signed all Google accounts and deleted browsing history. All searches and content extraction were conducted on February 22, 2025. Because website contents can be updated or modified over time, the information we analyzed reflects the versions available on that date.

### 2.2 Inclusion and exclusion criteria

The inclusion criteria were as follows: (i) written in Japanese, (ii) created by a medical institution, and (iii) accessible without a login or password. The exclusion criteria were as follows: (i) duplicate pages; (ii) pages created by government bodies, local municipalities, academic societies, or companies; and (iii) news articles or press releases. Government bodies and local municipalities were excluded because only hospitals provide detailed information, conduct examinations, and review the information shared by the entities performing these examinations. Additionally, we anticipated that patients would primarily refer to the websites of the medical institutions they intend to visit. For RQ1–RQ3, we included only webpages provided by institutions listed as certified by the Steering Committee [33]. For RQ4, since some non-certified institutions operate as corporate entities, we included companies, hospitals, and clinics not listed by the Steering Committee, using the same search terms, and extracted another set of 90 websites. The first author (JK) recorded the names and URLs of all websites, names of the medical institutions operating the websites, and certification statuses. JK reviewed all websites in the search results and extracted eligible websites. The names and URLs of all sites were documented on a Mac spreadsheet using Microsoft Excel.

### 2.3 Data assessment

First, the extracted websites were assessed by JK, a board-certified obstetrician and gynecologist. Comprehensiveness was evaluated using relevant guidelines, quality was assessed using the DISCERN instrument, and readability was assessed using jReadability [34]. Subsequently, the third author (RS), also a board-certified obstetrician and gynecologist, participated in a 1-h training session, after which RS independently evaluated 35% (12/34) of the websites for both comprehensiveness and quality. These 12 websites were randomly selected to generate random numbers using JMP Pro 17. The agreement of the 12 websites and inter-rater reliability between JK and RS were then assessed. We recorded all data in Microsoft Excel using a Mac spreadsheet by JK. The coding performed by JK was used for the analysis.

### 2.4 Evaluation criteria

#### 2.4.1 Comprehensiveness

Comprehensiveness was defined as the number and proportion of guideline recommendations covered by a website. We based the evaluation on three key documents: the “Guidelines for Prenatal Genetic Testing Using Maternal Blood (NIPT)” by the Ethics Committee of the Japan Society of Obstetrics and Gynecology, the “Recommendations on Prenatal Genetic Counseling” by the Japanese Society of Genetic Counseling, and “Information to Include on Your Website and Patient Leaflets About Non-Invasive Prenatal Testing (NIPT)” published by the UK Nuffield Council on Bioethics [[35], [36], [37]] (Appendix A). We compared the number and proportion of items addressed by each website to the total number of recommendations, and conducted 0/1 coding for agreement with these guidelines.

#### 2.4.2 Quality

The DISCERN instrument, developed by Deborah et al., is widely used to evaluate the reliability and quality of health information for patients [38,39]. It consists of 16 questions, and each DISCERN item is assessed on a 5-point Likert scale (1 = not at all, 5 = completely). Section 1 (Q1–Q7) assesses reliability, section 2 (Q8–Q15) evaluates the quality of information on treatment choices, and Q16 provides an overall rating. The DISCERN tool has been validated for both reliability and construct validity and has been translated and used in many countries, including its original English and Japanese versions [[40], [41], [42]]. Previous studies have demonstrated good inter-coder agreement when applying DISCERN to genetic testing websites, suggesting its utility for developing and assessing patient-directed resources [43]. The DISCERN scores range from 16 to 80, with higher scores indicating higher information quality. Scores <27, 27–38, 39–50, 51–62, and ≥ 63 indicate “very poor,” “poor,” “fair,” “good,” and “excellent” quality, respectively [44,45]. The coding criteria are attached as Appendix B.

#### 2.4.3 Readability

Readability was assessed using the Japanese version of jReadability [34]. This tool automatically evaluates readability based on average sentence length, word difficulty, distribution of parts of speech, and types of characters used per sentence [34,46]. For each website, we copied the main text into Microsoft Word for Mac, removed formatting elements that might interfere with readability scoring (e.g., headings, symbols, author information, and references) and reformated texts (e.g., set the space to the appropriate length), and then ran the text through jReadability. This system applies this regression formula to input text and returns a score in six-level categories shown in Table 1.

**Table 1:** Readability score categories.

Level	Ability
Lower beginner	Understand basic Japanese expressions centered on simple sentences. Cannot understand complex sentence structures such as compound sentences or attributive modifier structures.
Upper beginner	Understand basic vocabulary and grammar points. Understand basic compound sentences using conjugation.
Lower intermediate	Understand relatively straightforward writing and can grasp the content of moderately complex texts.
Upper intermediate	Understand the overall meaning of moderately technical texts and can easily understand everyday writing.
Lower advanced	Understand specialized texts with little difficulty and comprehend complex structures found in literary works.
Upper advanced	Understand highly specialized texts without difficulty. No trouble with any Japanese text.

#### 2.4.4 Non-certified institutions

For non-certified institutions, comprehensiveness, quality, and readability were assessed using the same criteria as certified institutions, and a comparison was conducted. However, because noncertified institutions were often part of larger corporate groups and the number of individual institutions was insufficient for statistical analysis, formal statistical comparisons were not performed.

### 2.5 Statistical analysis

Continuous variables were summarized using means and standard deviations (SDs), and categorical variables were summarized using frequencies and percentages. Inter-rater reliability was assessed using Cohen's kappa coefficient for the average number and proportion of comprehensive items and DISCERN scores with 95% confidence intervals obtained with bootstrap technique. Statistical analysis was performed using Python.

### 2.6 Ethical approval

This study did not involve human or animal participants, and all data were collected from publicly available online sources. This study was granted an exemption from ethics approval by the ethics review committee of the Graduate School of Medicine, University of Tokyo.

## 3 Results

**Fig. 1:**
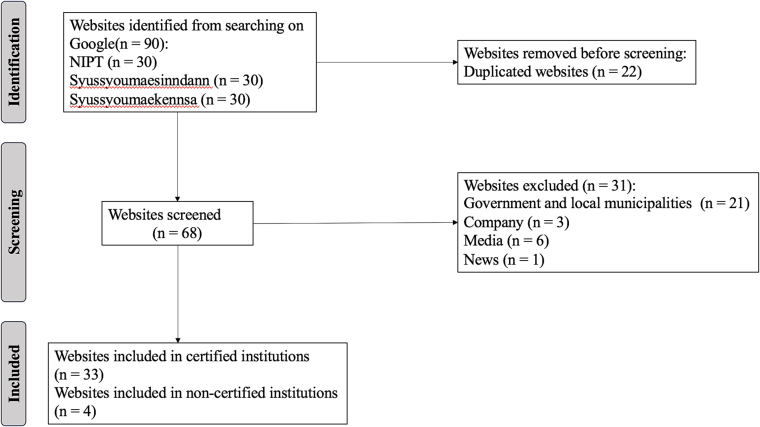
Search process flowchart. Note: This figure displays the analytic sample of the study (*N* = 37).

We identified ninety websites through a Google search. After removing duplicates and websites created by government bodies, media outlets, news services, and press releases, 37 websites remained for analysis. Among them, 33 websites were from certified institutions and 4 from non-certified institutions (Fig. 1). The number of non-certified institutions was low because they are operated as groups. A summary of the raw data for all results related to the certified institutions is provided in Appendix C.

### 3.1 Comprehensiveness

**Fig. 2:**
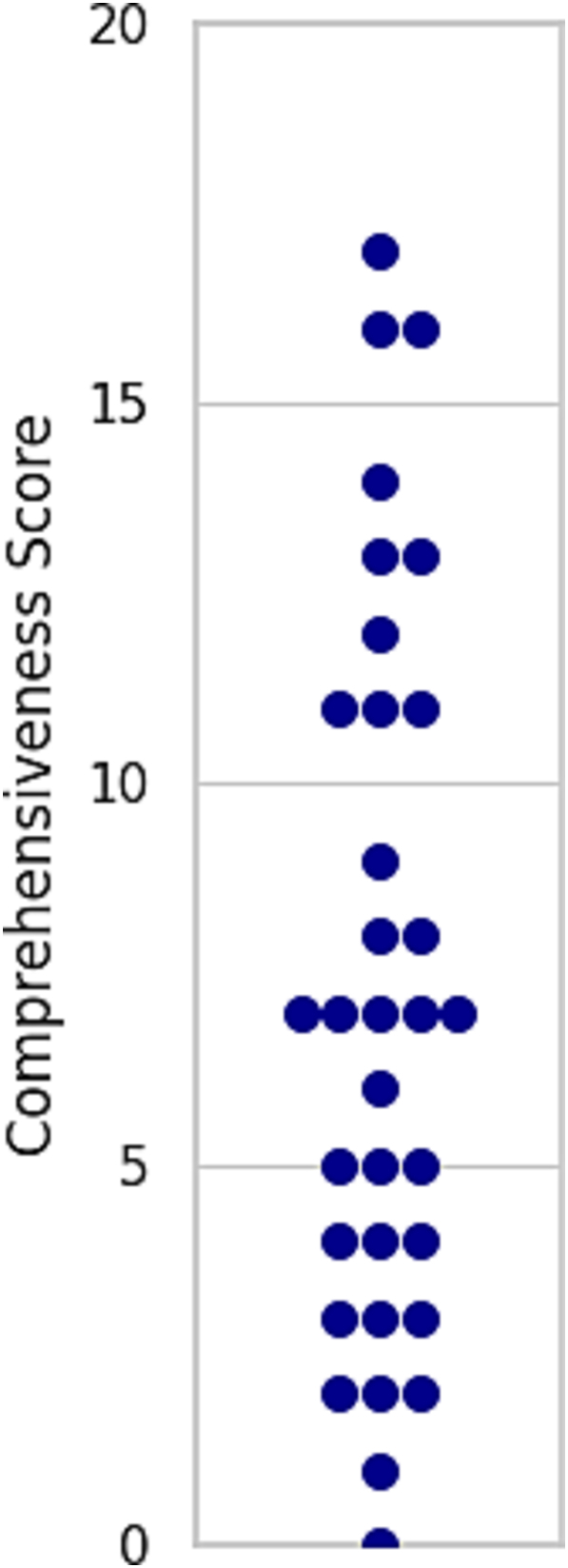
Plot of comprehensiveness scores by Facility. Note: This plot figure shows comprehensiveness scores by facility. The maximum score is 20. The average score of each facility is 8.09 (±4.40).

Fig. 2 and Table 2 present the comprehensiveness results for websites from certified institutions. The comprehensiveness of information is consistently low, indicating that the amount of information provided on many websites is insufficient. The inter-rater agreement was acceptable (average Cohen's κ = 0.635, 95% CI: 0.531–0.735). The mean (SD) comprehensiveness score for the 33 certified institutions was 7.36 (±4.70) 20. Only 10 websites (30.3%) scored >10, whereas 14 websites (42.4%) scored ≤5, indicating several websites with limited coverage. Items 3–6, 17, 19, and 20 were particularly underrepresented across the websites.

**Table 2:** Comprehensiveness scores for each question.

Question	Point (0−33)
Q1: Neutrality	29
Q2: Possibility of congenital anomalies	13
Q3: Possibility of disabilities after birth	2
Q4: Characteristics and symptoms of chromosomal abnormalities	6
Q5: Medical care for children with chromosomal abnormalities	5
Q6: Varieties of symptoms of chromosomal abnormalities	7
Q7: Be prepared for possible positive results	11
Q8: Target abnormalities of NIPT	27
Q9: Diseases not covered by NIPT in Japan	12
Q10: NIPT is a non-diagnostic screening test	10
Q11: Needs for taking invasive procedures	26
Q12: Possibility of false negative	11
Q13: Possibility of false positive	10
Q14: Possibility of not reportable	19
Q15: Roles of partner and core institutions	19
Q16: Risk of miscarriage associated with the invasive procedure	17
Q17: Possibility of complications caused by induced abortion	0
Q18: Options when result is not reportable	10
Q19: Options not taking confirmatory invasive testing	3
Q20: NIPT is not a guarantee of a “normal” outcome or merely a means to obtain reassurance.	6
Mean ± SD	12.15 ± 8.33

Note: Q3: In addition to congenital disabilities caused by prenatal factors, acquired disabilities may also develop after birth.

Q4: Characteristics and symptoms of chromosomal abnormalities targeted by NIPT (trisomy 21, 18, and 13).

Q5: Current state of medical care for children born with these chromosomal abnormalities, including the possibilities of treating complications and the availability of supportive care.

Q6: The prognosis of children born with these chromosomal abnormalities varies greatly among individuals; therefore, postnatal life experiences differ case by case.

Q17: If a pregnant woman decides to terminate the pregnancy, the procedure carries some complications.

Q19: The decision regarding whether or not to undergo confirmatory invasive testing should be made by the pregnant woman and her partner (including those in de facto marital relationships), based on sufficient genetic counseling regarding NIPT.

Q20: Since there are women who receive positive test results, it should be clearly stated that NIPT is not a guarantee of a “normal” outcome or merely a means to obtain reassurance.

### 3.2 Quality

**Fig. 3:**
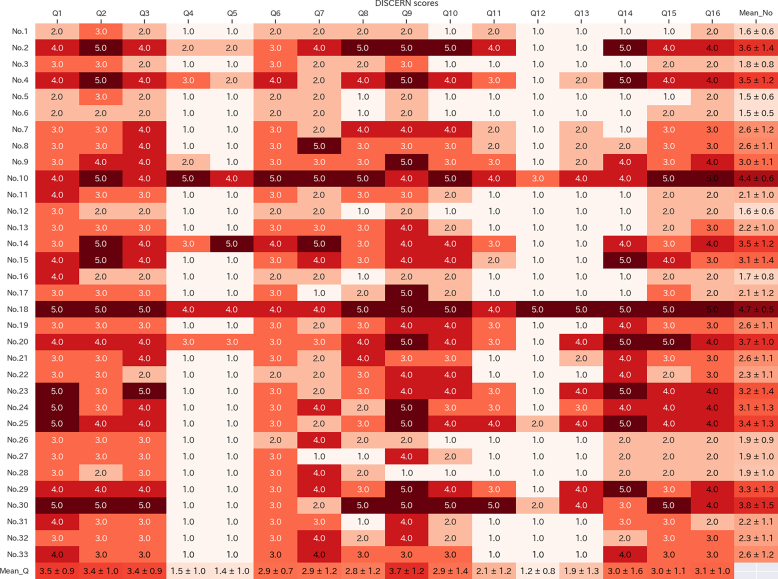
DISCERN scores for each facility. Note: This figure reflects the DISCERN scores for each facility in a heat map. Each DISCERN item is assessed on a 5-point Likert scale(1 = not at all, 5 = completely). The bottom row of the figure shows the mean and standard deviation for each score. The rightmost column shows the mean and standard deviation for each facility.

The results of the DISCERN evaluation for certified institutions are presented in Fig. 3. Although a small number of institutions provided acceptable information, the majority offered content of relatively poor quality. The inter-rater agreement was acceptable (average weighted Cohen's κ = 0.70, 95%CI: 0.613–0.774). The mean (SD) DISCERN score was 42.6 (±13.68). Two websites were rated as “excellent,” eight as “good,” eight as “fair,” eleven as “poor,” and four as “very poor.” Questions 4–8 and 10–14 had mean scores below 3, with questions 4 (citation sources), 5 (dates of references), 12 (benefits of not undergoing treatment), and 13 (impact on quality of life) scoring particularly low, often in the 1-point range, indicating significant deficiencies.

### 3.3 Readability

**Fig. 4:**
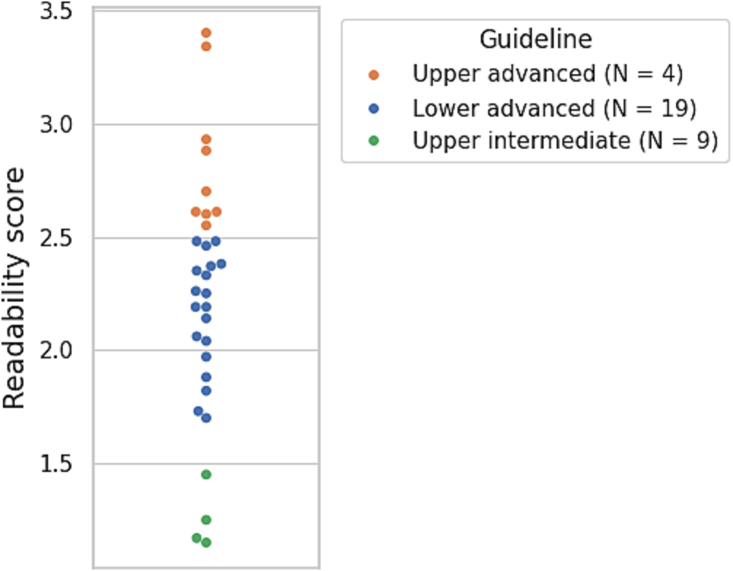
Plot of readability scores and guidelines by facility. Note: This dot plot figure displays readability scores and guidelines for each facility. The higher the score, the easier the Japanese text is to read (score range: 0–11.724). Since one facility could not be analyzed, this figure contains the results of 32 facilities. jReadability guideline classified each text into six readability levels (“lower elementary,” “upper elementary,” “lower intermediate,” “upper intermediate,” “lower advanced,” and “ upper advanced”).

The readability results for certified institutions are shown in Figs. 4. The mean readability score was 2.24 (±0.55). More than half of the websites were categorized as “lower advanced” or above, indicating that the reading level required comprehension of long, complex, and professional Japanese text.

### 3.4 Non-certified institution

Finally, Table 3 summarizes the results for the four websites of the non-certified institutions and Table 4 shows comparison between certified and non-certified institutions. Compared with certified institutions, non-certified websites had higher comprehensiveness scores, lower DISCERN scores, and higher readability scores. This means non-certified institutions provide more broader and readable information on their own websites.

**Table 3:** Scores for non-certified institutions.

Institutions	Comprehensiveness (0−20)	DISCERN (16–80)	Readability (0–11.724)	Categories
No. 34	9	38	2.73	Upper intermediate*
No. 35	8	37	3.04	Upper intermediate
No. 36	12	33	2.43	Lower advanced⁎⁎
No. 37	13	46	2.26	Lower advanced
Mean ± SD	10.5 ± 2.38	38.5 ± 5.45	2.66 ± 0.34	

Note: *Upper intermediate means that individuals are able to comprehend the overall meaning of moderately technical texts and can easily understand everyday writing.

⁎⁎ Lower advanced means that individuals are able to comprehend specialized texts with little difficulty and comprehend complex structures found in literary works.

**Table 4:** Comparison between certified and non-certified institutions.

Institutions	Comprehensiveness (0–20)	DISCERN (16–80)	Readability (0–11.724)
Certified	12.15 ± 8.33	42.6 ± 13.68	2.24 ± 0.55
Non-certified	10.5 ± 2.38	38.5 ± 5.45	2.66 ± 0.34

## 4 Discussion and conclusion

### 4.1 Discussion

This study evaluated the comprehensiveness, quality, and readability of information on the NIPT provided by medical institutions' websites. The proportion of websites from certified institutions that achieved comprehensiveness was low, the overall quality was insufficient, and the readability level often required readers to comprehend complex Japanese texts. The comparison revealed a paradox between certified and non-certified institutions; in some cases, non-certified institutions provided more comprehensive, readable, and even higher-quality information than certified ones while the text lacked informational integrity, with inaccuracies and exaggerated expressions. Consequently, such situations in online information may undermine patient understanding, compromise the quality of decision-making, and contribute to the diversion of patients to non-certified institutions without genetic counseling. These findings suggest that, rather than focusing solely on the quantity or apparent quality of information, it is important to provide accurate, well-founded, and easy-to-read information from a neutral standpoint.

Beyond documenting these descriptive patterns, this study makes three contributions to the broader literature. First, by jointly evaluating comprehensiveness, quality, and readability, it demonstrates how multiple dimensions of online information interact to shape the accessibility of prenatal testing information—a relationship that has rarely been empirically examined in prior work. Second, the misalignment between certification status and information quality provides novel evidence that institutional credibility does not guarantee effective communication, highlighting a structural gap that can undermine patients' decision-making. Third, our findings carry international significance by illustrating how commercial providers, even outside formal regulatory frameworks, may outperform certified institutions in the usability of web-based materials. These dynamic echoes concerns raised in other countries regarding the growing role of commercial actors in prenatal testing markets and underscores the need for global dialogue on organizational health literacy and equitable information provision.

### 4.2 Comprehensiveness

Many certified institutions failed to achieve informational sufficiency on their websites. Although websites are not a source of comprehensive patient education, many lack basic information. Particularly, studies regarding the potential for acquired disabilities, explanations of chromosomal disorders, information on postnatal social support, the risks associated with termination of pregnancy, the voluntary nature of confirmatory testing, and the notion that NIPT does not guarantee a “normal” result or “reassurance” are lacking. This may lead patients to develop misconceptions about NIPT and may amplify ambivalent emotions [47]. Certified institutions may be deliberately avoiding disclosure of information to prevent discussions about disabilities from veering off course, and this could be a reason for the lack of transparency. Pre-exposure to comprehensive and accurate information via websites can improve patient understanding [47]. Knowledge and the provision of appropriate information are important factors in the quality of decision-making regarding NIPT.

### 4.3 Quality

Information must not only be sufficient but also of high quality. Regarding the quality, the overall mean rating of websites was categorized as “fair,” although approximately half of websites were rated as “poor” or “very poor.” Analysis of individual DISCERN instruments revealed low scores for questions 4, 5, 12, and 13 and relatively high scores for questions 1, 2, and 9, which is consistent with previous studies [[48], [49], [50]]. When explaining NIPT, the use of numerical data, such as positive predictive values and maternal age-related risk, necessitates a clear citation of sources, which is often missing. Furthermore, the psychological impact of deciding whether to undergo testing and awaiting test results should be discussed, including the possibility of worsened quality of life; however, such mentions are rare [47]. Accurate and high-quality information would reduce women's perception of decisional conflict [51]. However, lack of citations and failure to address quality of life impacts undermine credibility and limit support for effective SDM. Healthcare institutions should use reliable sources and clearly state how testing affects daily life to enable better decisions.

### 4.4 Readability

The Japanese version of the readability assessment considers both sentence length and the proportion of kanji characters. Although Japan has an almost 100% literacy rate and high global reading ability, kanji characters can sometimes be challenging to read, leading to increased time needed for text comprehension. Most websites related to NIPT require at least an upper-intermediate reading proficiency. This indicates that individuals at this level can typically comprehend moderately technical content and navigate everyday writing with ease. This finding is consistent with those of previous reports showing that websites published by universities and public institutions in Japan have higher readability levels than those published by other types of institutions [52]. Previous studies have indicated that reading ability is associated with health outcomes, highlighting the importance of easily understood methods for improving patient comprehension [53]. Even in face-to-face consultations, failure to define medical terminology can negatively affect patient comprehension and SDM [54]. Given that NIPT requires patients to understand complex genetic information within a limited amount of time, prior exposure to high-quality information through websites could potentially improve comprehension [55]. Because NIPT-related information contains complex medical terms, the inclusion of narratives and visual aids, such as diagrams and illustrations, may make the content more accessible and easier to understand.

### 4.5 Non-certified institutions

Websites from non-certified institutions often included exaggerated expressions and unbalanced information and overstated the reliability of experimental tests that raised ethical concerns; however, these sites also tended to provide more detailed information regarding conditions such as Down syndrome and postnatal support compared to websites from certified institutions. Unlike certified institutions, many of these non-certified sites were relatively comprehensive and presented information in a reader-friendly manner. These were because they wanted to enhance patient attraction by presenting comprehensive and easy-understandable materials, and to increase profits by actively recommending examinations. Approximately 70% of non-certified institutions provide only brief oral explanations or even omit oral explanations entirely, relying solely on written materials [27]. Furthermore, many non-certified institutions are operated by administrators who are not obstetricians or gynecologists. Receiving an inadequate information at non-certified institutions can lead to patients undergoing NIPT without sufficient understanding or developing misconceptions regarding the testing. This undermines the process of SDM and can increase patients' psychological burdens and conflicts.

### 4.6 Limitations

This study had some limitations. First, the sample size was small. As many non-certified institutions operate under group structures, the number of institutions included was relatively smaller than the actual number of institutions, making it difficult to achieve sufficient statistical power. Second, the study targeted only the top 30 search results for each keyword on Google, thus inducing a potential selection bias. However, because most Internet users typically access only the first few pages of search results, the findings are likely to be a valid representation in a practical situation. Third, only Japanese websites were included, limiting the generalizability of the results to other linguistic and cultural contexts. Although the presence of non-certified institutions is a limited problem specific to Japan, it is comparable to issues observed internationally regarding the provision of information by commercial entities. Fourth, the authors selected the comprehensive evaluation items arbitrarily, which may have limited their external validity. Nevertheless, the primary author and second coder was a board-certified obstetrician-gynecologist, providing some assurance of content validity. Fifth, application of the DISCERN tool involved subjective, unblinded assessments, raising the chance of bias. Sixth, Since the evaluation of each institution was not conducted in a blinded manner, there is a possibility of bias. Seventh, we did not include images or infographics in the readability assessment. Because the readability system cannot evaluate these materials, these results do not enable us to make a direct comparison of the understandability of the websites and materials. Finally, improving the quality of websites may not directly lead to appropriate uptake behavior regarding NIPT.

### 4.7 Innovation

To the best of our knowledge, this is the first study to systematically evaluate the content of NIPT-related information published on websites operated by medical institutions in Japan. While many patients obtain health information from the internet, there has been little empirical research on the content and accessibility of NIPT information provided by healthcare institutions. Our findings indicate that institutional online communication often demands advanced literacy skills while providing limited support for genuine understanding. This challenges current assumptions about the adequacy of institutional information provision and suggests practical directions for improvement. Additionally, by comparing certified and non-certified institutions in Japan's Prenatal Testing Certification System, this study shows clear differences in how information is provided, which may influence patients' perception of NIPT and their decision-making. These findings offer practical insights into institutional communication gaps and the potential behavioral implications for patients navigating NIPT choices. Although this system is specific to Japan, the basic tension between regulation, commercial interests, and clear communication is similar to problems seen in many countries when health information is provided. Given the anticipated growth in demand for NIPT even amidst declining birth rates, our study highlights the need to reconsider how prenatal screening information is communicated in digital environments. Because time during genetic counseling sessions is limited, patients may struggle to fully grasp all the information presented. Accessing high-quality information in advance can help enhance their understanding. Approaches that incorporate patient-centered perspectives, such as co-designing online informational materials with patients, may help bridge the gap between technical accuracy, ethical responsibility, and practical comprehensibility.

## 5 Conclusion

This study evaluated the comprehensiveness, quality, and readability of NIPT-related information provided on the websites of medical institutions in Japan. We identified a central paradox: certified institutions, despite their formal credibility and regulatory legitimacy, often provide less comprehensive and less readable information than non-certified institutions, which tend to offer more detailed but potentially biased or ethically problematic content. From an SDM perspective, unclear or insufficiently accessible information from certified institutions may unintentionally steer patients toward non-certified providers, where exaggerated or misleading claims may influence decision-making. Website quality should therefore be understood not merely as a technical or educational matter, but as an ethical responsibility of healthcare institutions, directly linked to patient autonomy and informed choice. These findings underscore the need for both stronger oversight of non-certified institutions and proactive efforts by certified institutions to improve the informational sufficiency, quality, and readability of their online information. Future research should include mixed-method follow-up studies combining content analysis with patient interviews to evaluate the real-world effects of online information, as well as longitudinal monitoring to determine whether recent policy changes or certification reforms improve information quality.

## Funding sources

This work was supported by the 10.13039/501100001691Japan Society for the Promotion of Science KAKENHI (25 K13483).

## CRediT authorship contribution statement

**Junya Kohari:** Writing – original draft, Project administration, Methodology, Formal analysis. **Tsuyoshi Okuhara:** Writing – review & editing, Validation, Supervision, Methodology. **Ritsuko Shirabe:** Writing – review & editing, Formal analysis.

## Declaration of generative AI and AI-assisted technologies in the writing process

During the preparation of this work the author(s) used ChatGPT in order to refine my English writing. After using this tool/service, the author(s) reviewed and edited the content as needed and take(s) full responsibility for the content of the publication.

## Declaration of competing interest

The authors declare that they have no competing financial interests or personal relationships that may have influenced the work reported in this study.

## Data Availability

The data that support the findings of this study are available from the corresponding author, JK, upon reasonable request.

## Appendix A Supplementary data

Supplementary material 1 Image 1

Supplementary material 2 Image 2

Supplementary material 3 Image 3
