# Supplementary material for: Comprehensiveness, quality, and readability of non-invasive prenatal testing information on Japanese medical institution websites
Source: PEC Innov. 2026 Feb 22;8:100462. doi: 10.1016/j.pecinn.2026.100462 (PMC12966698; doi:10.1016/j.pecinn.2026.100462)
Supplement: Supplementary file 1 — Supplementary material 1 [file mmc1.docx]

# Appendix: Informational Items Regarding NIPT(Comprehensiveness)

1. Information should be provided from a neutral standpoint regarding whether or not to undergo NIPT, and must not guide toward either undergoing or not undergoing the test.
2. Every child has the possibility of being born with congenital anomalies or disabilities, and these possibilities vary widely.
3. In addition to congenital disabilities caused by prenatal factors, acquired disabilities may also develop after birth.
4. Characteristics and symptoms of chromosomal abnormalities targeted by NIPT (trisomy 21, 18, and 13).
5. Current state of medical care for children born with these chromosomal abnormalities, including the possibilities of treating complications and the availability of supportive care.
6. The prognosis of children born with these chromosomal abnormalities varies greatly among individuals; therefore, postnatal life experiences differ case by case.
7. Any couple may have a chance of conceiving a fetus with a chromosomal numerical abnormality; thus, individuals should be mentally prepared for a possible positive result before undergoing the test.
8. Although 60–70% of chromosomal abnormalities detected by invasive diagnostic procedures are numerical abnormalities, NIPT only targets trisomy of chromosomes 21, 18, and 13.
9. Current NIPT in Japan does not target other abnormalities such as balanced translocations, microdeletions or duplications, fetal chromosomal mosaicism, confined placental mosaicism, or single-gene disorders.
10. Although NIPT aims to detect numerical abnormalities in chromosomes 21, 18, and 13, it is a non-diagnostic screening test that indicates a high likelihood but does not confirm a diagnosis. The positive predictive value varies with maternal age, and false-positive rates tend to be higher among younger women.
11. A definitive diagnosis of numerical abnormalities in chromosomes 21, 18, and 13 requires invasive procedures such as chorionic villus sampling (CVS) or amniocentesis.
12. A negative test result means that the likelihood of having one of the target chromosomal abnormalities is extremely low but not zero; false negatives are possible. Therefore, a negative result does not confirm the absence of the targeted abnormalities.
13. A positive result increases the likelihood of one of the target chromosomal abnormalities being present, but false positives can occur. The positive predictive value depends on the prior probability. A definitive diagnosis requires confirmatory invasive testing (CVS or amniocentesis).
14. There are cases in which the test result may be inconclusive (Not Reportable).
15. When a positive result is obtained through a partner institution, post-test genetic counseling should, in principle, be conducted at the affiliated core institution. Furthermore, depending on the pregnant woman’s wishes, follow-up—including confirmatory invasive testing—may also be conducted at the core institution.
16. Confirmatory invasive testing using amniocentesis involves chromosomal testing of fetal cells obtained from amniotic fluid, and there is approximately a 1 in 300 risk of miscarriage associated with the procedure.
17. If a pregnant woman decides to terminate the pregnancy based on the results of confirmatory invasive testing, the procedure carries some complications.
18. In the event of an inconclusive (Not Reportable) result, options include re-testing or proceeding directly to confirmatory invasive testing.
19. The decision regarding whether or not to undergo confirmatory invasive testing should be made by the pregnant woman and her partner (including those in de facto marital relationships), based on sufficient genetic counseling regarding NIPT.
20. Since there are women who receive positive test results, it should be clearly stated that NIPT is not a guarantee of a “normal” outcome or merely a means to obtain reassurance.
