# Supplementary material for: Comprehensiveness, quality, and readability of non-invasive prenatal testing information on Japanese medical institution websites
Source: PEC Innov. 2026 Feb 22;8:100462. doi: 10.1016/j.pecinn.2026.100462 (PMC12966698; doi:10.1016/j.pecinn.2026.100462)
Supplement: Supplementary file 2 — Supplementary material 2 [file mmc2.docx]

DISCERN Criteria

Section 1 – Reliability of the publication

Question 1: Are the aims clear?
Assess whether the publication clearly states at the beginning:
Criteria 1: The aim is stated within the first one or two paragraphs of the website.
Criteria 2: The aim of the page (e.g., providing information about NIPT or explaining the testing process) is explicitly described.
Criteria 3: The intended audience is clearly specified (e.g., “for individuals considering NIPT”).

Scoring:
5 — All three criteria are met, and both the purpose and target audience are clearly identified.
4 — Criteria 2 and 3 are met and presented early in the website (not necessarily in the first paragraphs).
3 — Either criterion 2 or 3 is unclear.
2 — Either criterion 2 or 3 is absent.
1 — The purpose of the publication is unclear.

Question 2: Does it achieve its aims?
After reviewing the website as a whole, assess whether the information provided corresponds to the stated aim (provision of information regarding NIPT).

Scoring:
5 — Information fully consistent with the aim is provided.
4 — Information is largely consistent with the aim.
3 — Information is relevant but incomplete.
2 — Information is not consistent with the aim.
1 — Information is inconsistent with the aim and may contain misleading content.

Question 3: Is it relevant?
Assess whether the content includes information necessary for informed decision‑making among pregnant women considering NIPT and their families, and whether the tone is supportive and patient‑centered.

Scoring:
5 — Provides balanced, decision‑relevant information expressed in a supportive manner.
4 — Provides largely balanced information, but lacks a supportive tone.
3 — Provides some decision‑relevant information, but is incomplete or biased and not supportive.
2 — Provides limited or biased information and lacks a supportive tone.
1 — Not useful for individuals in this situation.

Question 4: Is it clear what sources of information were used?
Determine whether key factual statements are supported by citations to research articles or expert opinions (excluding “further reading” lists). References should preferably be compiled at the end of the text.

Scoring:
5 — Nearly all key information is referenced (>90%).
4 — Partial citation coverage (70–90%).
3 — Moderate citation coverage (40–60%).
2 — Limited citation coverage (10–40%).
1 — No or almost no citations.

Question 5: Is it clear when the information used was produced?
Assess whether dates are provided for cited information. Note that a low score on Question 4 limits the maximum possible score here.

Scoring:
5 — Dates are provided for nearly all citations (>90%).
4 — Dates are provided for most citations (70–90%).
3 — Dates are provided for approximately half (40–60%).
2 — Dates are provided for a few citations (10–40%).
1 — Citations rarely include dates.

Question 6: Is it balanced and unbiased?
Evaluate whether the text presents information from a neutral perspective without sensationalist expressions or alarming statistics, and whether content is evidence‑based. A low score on Question 4 limits the maximum possible score here.

Scoring:
5 — Balanced and appropriate language supported by multiple sources of evidence.
4 — Balanced expression with limited evidence referencing.
3 — Somewhat balanced or minor issues in tone, with limited evidence.
2 — Noticeable imbalance in tone and insufficient evidence.
1 — Highly biased or inappropriate expression with almost no evidence to support.

Question 7: Does it provide details of additional sources of support and information?
Determine whether further reading materials or consultation contacts are listed with sufficient contact details.

Scoring:
5 — Both further reading materials and consultation contacts are provided.
4 — Further reading materials or consultation contacts are insufficient.
3 — Both further reading materials and consultation contacts are insufficient.
2 — Further reading materials or consultation contacts are absent.
1 — Neither further reading materials nor consultation contacts are provided.

Question 8: Does it refer to areas of uncertainty?
Assess whether gaps in knowledge, differences in expert opinion, or uncertainty of outcomes are acknowledged.

Scoring:
5 — Uncertain issues are clearly identified, explained, and contextualized.
4 — Most uncertainties are identified and explained.
3 — Insufficient discussion of uncertainties.
2 — Minimal mention of uncertainties.
1 — No mention or misleading description of uncertainties.

Section 2 – Quality of treatment information

Question 9: Does it describe how each test works?
For each test described on the website, determine whether procedures and objectives are clearly explained.

Scoring:
5 — Procedures and objectives are clearly explained for all tests.
4 — Procedures and objectives are mostly explained.
3 — Explanations are present but incomplete.
2 — Either procedures or objectives are missing or insufficient.
1 — Neither procedures nor objectives are described.

Question 10: Does it describe the benefits of each treatment?

Confirm whether benefits are clearly described for each test listed.

Scoring:
5 — Benefits for all tests are clearly stated.
4 — Benefits are stated but somewhat incomplete.
3 — Benefits described for some tests only.
2 — Benefits are rarely described.
1 — No benefits described.

Question 11: Does it describe the risks of each treatment?

Risks include medical and ethical considerations.

Scoring:
5 — Risks for all tests are clearly described.
4 — Risks for most tests are described.
3 — Risk descriptions are present but incomplete.
2 — Risk descriptions are insufficient.
1 — No risk descriptions are provided.

Question 12: Does it describe what would happen if no test is used?
Assess whether fair descriptions of the advantages and disadvantages of not undergoing NIPT are provided.

Scoring:
5 — Advantages and disadvantages are explained comprehensively.
4 — Advantages and disadvantages are explained adequately.
3 — Explanations are present but incomplete.
2 — Explanations are minimal.
1 — No explanations are provided.

Question 13: Does it describe how the treatment choices affect overall quality of life (QoL)?
Assess discussion of lifestyle impact, emotional wellbeing, family and social effects.

Scoring:
5 — Both potential benefits and harms to QoL are comprehensively addressed.
4 — Benefits and harms to QoL are mostly addressed.
3 — Discussion is present but incomplete.
2 — Either benefits or harms to QoL are missing.
1 — No discussion or misleading description of QoL impact.

Question 14: Is it clear that there may be more than one possible test choice?
Determine whether options other than NIPT (e.g., first‑trimester ultrasound screening, combined testing) are described.

Scoring:
5 — Two or more alternatives are presented with detailed explanations.
4 — Two or more alternatives are presented but details are insufficient.
3 — Alternatives are mentioned but lack sufficient detail.
2 — Only one alternative is mentioned or poorly described.
1 — No alternatives are mentioned.

Section 3 – Overall evaluation

Question 15: Does it provide support for shared decision‑making?
Assess whether the publication helps readers identify issues to discuss with clinicians, family, or carers.

Scoring:
5 — Excellent
4 — Good
3 — Fair
2 — Poor
1 — Bad

Question 16: Overall quality rating

Overall intuitive assessment considering all criteria:

5 — Excellent
4 — Good
3 — Fair
2 — Poor
1 — Bad
