# Supplementary material for: Comprehensiveness, quality, and readability of non-invasive prenatal testing information on Japanese medical institution websites
Source: PEC Innov. 2026 Feb 22;8:100462. doi: 10.1016/j.pecinn.2026.100462 (PMC12966698; doi:10.1016/j.pecinn.2026.100462)
Supplement: Supplementary file 3 — Supplementary material 3 [file mmc3.docx]

**Appendix C**

Scores of each institution

| institution | Comprehensiveness | DISCERN | Readability |
| --- | --- | --- | --- |
| No.1 | 2 | 26 | Unmeasurable |
| No.2 | 11 | 58 | 2.46 |
| No.3 | 3 | 29 | 2.33 |
| No.4 | 17 | 56 | 1.88 |
| No.5 | 2 | 24 | 2.6 |
| No.6 | 1 | 24 | 1.17 |
| No.7 | 12 | 41 | 1.73 |
| No.8 | 5 | 42 | 3.4 |
| No.9 | 11 | 48 | 2.06 |
| No.10 | 14 | 71 | 2.25 |
| No.11 | 9 | 33 | 2.48 |
| No.12 | 6 | 25 | 2.38 |
| No.13 | 4 | 35 | 1.82 |
| No.14 | 16 | 56 | 2.88 |
| No.15 | 13 | 49 | 2.48 |
| No.16 | 2 | 27 | 3.34 |
| No.17 | 5 | 33 | 1.25 |
| No.18 | 16 | 75 | 1.97 |
| No.19 | 5 | 42 | 1.15 |
| No.20 | 7 | 59 | 1.7 |
| No.21 | 11 | 41 | 2.37 |
| No.22 | 0 | 37 | 2.7 |
| No.23 | 8 | 52 | 2.26 |
| No.24 | 8 | 50 | 1.45 |
| No.25 | 7 | 55 | 2.14 |
| No.26 | 3 | 31 | 2.61 |
| No.27 | 4 | 31 | 2.61 |
| No.28 | 4 | 30 | 2.35 |
| No.29 | 7 | 53 | 2.19 |
| No.30 | 13 | 60 | 2.93 |
| No.31 | 3 | 36 | 2.19 |
| No.32 | 7 | 37 | 2.55 |
| No.33 | 7 | 41 | 2.04 |
